# The kynurenine derivative 3-HAA sensitizes hepatocellular carcinoma to sorafenib by upregulating phosphatases

**DOI:** 10.7150/thno.59841

**Published:** 2021-04-03

**Authors:** Guifang Gan, Zhaopeng Shi, Chengfang Shangguan, Jieying Zhang, Yuan Yuan, Lei Chen, Weiren Liu, Biao Li, Songshu Meng, Wujun Xiong, Jun Mi

**Affiliations:** 1 Basic Medical Institute; Hongqiao International Institute of Medicine, Tongren Hospital; Key Laboratory of Cell Differentiation and Apoptosis of Chinese Ministry of Education, Shanghai Jiao Tong University School of Medicine, Shanghai 200025, China.; 2 Department of Nuclear Medicine & Department of Oncology, Rui Jin Hospital, Shanghai Jiao Tong University School of Medicine, Shanghai 200025, China.; 3 Shanghai Ninth People's Hospital, College of Stomatology, Shanghai Jiao Tong University School of Medicine, Shanghai, 200011, China.; 4 Translational Medical Center for Stem Cell Therapy, East Hospital, Tongji University School of Medicine, 150 Jimo Road, Shanghai 200120, China.; 5 International Co-operation Laboratory on Signal Transduction, Eastern Hepatobiliary Surgery Institute, Marine Military Medical University, Shanghai 200438, China.; 6 Department of Liver Surgery, Liver Cancer Institute, Zhongshan Hospital, Fudan University, Shanghai 200032, China.; 7 Institute of Cancer Stem Cell, Dalian Medical University, Dalian, Liaoning 116044, China.; 8 Department of Gastroenterology, Shanghai Pudong Hospital, Fudan University Pudong Medical Center, Shanghai 201399, China.

**Keywords:** 3-hydroxyanthranilc acid, sorafenib resistance, AKT, DUSP6, PPP1R15A.

## Abstract

**Objectives**: Sorafenib is the only FDA-approved first-line target drug for HCC patients. However, sorafenib merely confers 3-5 months of survival benefit with less than 30% of HCC patients sensitive to sorafenib therapy. Thus, it's necessary to develop a sensitizer for hepatocellular carcinoma (HCC) to sorafenib. **Methods**: The principal component analysis, gene ontology, and KEGG analysis are utilized following RNA-sequencing. The mass spectrometry analysis following immunoprecipitation is performed to discover the phosphatase targets. Most importantly, both the cell line-derived xenograft (CDX) and the patient-derived xenograft (PDX) mouse model are used to determine the effect of 3-HAA on sorafenib-resistant HCC *in vivo*. **Results**: In nude mice carrying HCC xenograft, tumor growth is inhibited by sorafenib or 3-HAA alone. When used in combination, the treatment particularly prevents the xenograft from growing. Combined treatment also suppresses the growth of sorafenib-resistant (≥30mg/kg) PDXs. In a set of mechanistic experiments, we find enhanced AKT activation and decreased apoptotic cells in *de novo* and acquired sorafenib-resistant HCC cells and tissues. 3-HAA decreases AKT phosphorylation and increases the apoptosis of HCC in both cultured cells and mouse xenografts by upregulation of phosphatases PPP1R15A/DUSP6. PPP1R15A/PPP1α directly reduces Akt phosphorylation while DUSP6 decreases Akt activity through inhibiting PDK1. The AKT activator abolishes 3-HAA inhibition of HCC growth *in vitro* and in mice. **Conclusion**: This study demonstrates that 3-HAA sensitizes HCC cells to sorafenib by upregulation of phosphatases, suggesting it as a promising molecule for HCC therapy.

## Introduction

Hepatocellular carcinoma (HCC) is the fifth most prevalent primary cancer and the third leading cause of cancer-related death worldwide 1. Most HCC patients are at the advanced stage upon diagnosis and ineligible for surgical resection, liver transplantation or curative ablations. Even in patients receiving curative resection, approximately 70% of patients develop tumor recurrence within 5 years 2, 3. Sorafenib is the only FDA-approved first line tyrosine kinase inhibitor for HCC patients, especially those patients with recurrent or advanced HCC.

Sorafenib inhibits angiogenesis and induces apoptosis of HCC cells 4. Sorafenib targets the Raf/MAPK (mitogen-activated protein kinase)/ERK (extracellular signaling-regulated kinase) signaling pathway, and inhibits a number of tyrosine kinase receptors, including VEGF receptor, platelet-derived growth factor receptor, and *c*-Kit. However, sorafenib only confers 3-5 month survival benefit with less than 30% HCC patients sensitive to sorafenib therapy 5.

The mechanisms underlying HCC resistance to sorafenib are still not elucidated 6. Consistent activating survival signal and inhibiting apoptosis have been proposed as a critical mediator of resistance to antitumor therapies in advanced HCC 7. Current chemotherapies for advanced HCC could effectively reduce the part of tumor, but HCC cells with additional survival pathways could survive, resulting in a transient tumor volume reduction with a quick recurrence. Thus, targeting the critical mediator of compensatory pathways is a promising approach as it could circumvent the development of sorafenib resistance and synergizes with sorafenib to suppress HCC.

The phosphoinositide 3-kinase (PI3K)/AKT pathway regulates a large number of molecules involving in cancer progression and apoptosis, including HCC. Our current study shows that the other upstream pathways other than the tyrosine kinase receptor (RTK) signal activate AKT signaling and the AKT phosphorylation levels increase in sorafenib-resistant HCC cells and tissues, which is consistent with previous finding 8. This acquired resistance of HCC to sorafenib could involve compensatory activation of specific signaling pathways 6, 9. AKT activation promotes tumor cell proliferation and inhibits apoptosis 10, 11. Inhibition of AKT activity reverses acquired resistance of HCC to sorafenib 12, 13. Moreover, PI3K/AKT signaling has crosstalk with MAPK/ERK signaling pathways 14, indicating that latent compensatory activation of the PI3K/AKT pathway may contribute to sorafenib resistance of HCC.

Kynurenine, a catabolite of tryptophan, promotes immune evasion and tumor growth and the kynurenine/AHR signal has been implied as a contributory mechanism to cancer resistance 15, 16. Our data (Shi, et al. Kynurenine derivative 3-HAA is an agonist ligand for transcript factor YY1) showed 3-hydroxyanthranilic acid (3-HAA), a structural analog of kynurenine, was selectively downregulated in HCC cells and exogenous 3-HAA promoted apoptosis in HCC *in vitro and in vivo* via binding with transcription factor YY1. We speculate that adding 3-HAA to sorafenib therapy for HCC may help overcome sorafenib resistance. In this study, we employed 3-HAA to investigate whether 3-HAA could sensitize HCC cells and xenografts to sorafenib.

## Methods

### Cells

Human HCC cell line HepG2 (American Type Culture Collection; Manassas, VA, USA; RRID: CVCL_0027), Hep3B (RRID: CVCL_0326), PLC8024 (RRID: CVCL_0485) (Cell Bank of the Chinese Academy of Science, Shanghai, China) and SMMC7721 (RRID: CVCL_0336) (Genechem Co., Ltd., Shanghai, China) were grown at 37 °C in DMEM (Invitrogen, Grand Island, NY, USA) containing 5% CO_2_ atmosphere and supplemented with 10% heat-inactivated fetal bovine serum (PAA, Australia) in the presence of 100 U/mL penicillin and 0.1 mg/mL streptomycin.

### CCK8 assays

HCC cells were seeded into a 96-well plate at 2000 cells/well, were treated with 3-HAA (Sigma, St Louis, MO, USA) and/or sorafenib (Meilunbio Co., Ltd, Dalian, China) at appropriate doses as indicated elsewhere. BCI (#B4313) (Sigma) and SC79 and MK2206 (Selleck, Shanghai, China) were added as described elsewhere in the text. CCK8 assays were performed in triplicates as instructed by the manufacturer (Dojindo, Tabaru, Japan) for 2 h at indicated time points. Absorbance was measured at 450 nm using a microplate reader and cell viability was normalized to control and the mean of at least three independent experiments was calculated. In addition, the half maximal inhibitory concentration (IC_50_) was obtained of 3-HAA and/or sorafenib for each cell line.

### Phosphorylation antibody microarray assay

HCC cells were lysed by lysis beads in extraction buffer containing protease inhibitors and phosphatase inhibitors. The filtered protein solutions were labeled by biotin for 2 h, followed by incubating with phosphorylation antibody microchip in coupling solution for 2 h (PEX100, Full Moon Inc., USA). After 20 min incubation with Cy3-streptavidin solution, the microchips were scanned with the scanner (GenePix 4000B, Axon Instruments, USA). The data were analyzed by GenePix software (GenePix Pro 6.0, Axon Instruments, USA).

### Establishment of sorafenib-resistant HCC cells

Sorafenib-resistant clones of HepG2 and Hep3B cells were established by subjecting HCC cells to continuous administration of gradually increasing sorafenib concentrations and were trained up to 8µM (HepG2) and 10µM (Hep3B). The same volume of dimethyl sulf-oxide (DMSO) was added to the cells as mock controls during establishment of these resistant cells. Initially, we examined the 50% inhibiting concentration (IC50) of HepG2 and HepG2 cells following sorafenib treatment. Subsequently, we seeded HepG2 and Hep3B cells in six-well plates at a density of 1 × 10^4^ cells per well and incubated the cells with sorafenib concentrations just below their respective IC50. During the following time periods, the concentration of sorafenib was slowly increased by 0.25 µM per week. Following a 6-month treatment period, the two cell lines resistant to sorafenib were established and were named HepG2 SorR and Hep3B SorR. The cells were continuously maintained by culturing them in the presence of sorafenib. Finally, we incubated HepG2, Hep3B, HepG2-SorR, and Hep3B-SorR cells with gradually increasing doses of sorafenib in 96-well plates and determined the cell viability by the CCK8 assay following 2 days of incubation.

### Xenograft assays

Four-week-old male BALB/c nude mice were obtained from Shanghai SLAC Laboratory Animal Center, Shanghai, China. All mice were maintained according to the Guide for the Care and Use of Laboratory Animals published by the National Institutes of Health, USA. SMMC7721 cells (1×10^6^ cells) were injected subcutaneously into the armpit area of each mouse. When the tumor volume reached 80 mm^3^, 100 mg/Kg.day 3-HAA, 10 mg/Kg.day sorafenib and 40 mg/Kg.day SC79 was administered intraperitoneally daily for 7 days. The tumor volume was determined at the indicated time points using digital caliper measurements and calculated by the following formula:

Tumor volume (mm^3^) = ½×longest diameter^2^ × shortest diameter

At the indicated time points, the mice were sacrificed and the tumors were excised and tumor weight was measured.

The study protocol was approved by the Institutional Animal Care and Use Committee of Shanghai Jiao Tong University School of Medicine and animal study was carried out in accordance with established national and institutional guidelines on the use of experimental animals.

### HCC-PDX models

This study received ethics board approval at the Shanghai Jiao Tong University School of Medicine. The three HCC-PDX models (LIV#031, 045, 109) were originally isolated from sorafenib-resistant patients (> 30mg/kg.day), and were stored in liquid nitrogen. Mice were maintained under specific-pathogen-free (SPF) conditions. Once the recovered tumors grew to 250 mm^3^ in mice, tumor tissues were cut into 2×2 mm pieces and implanted subcutaneously into SCID mice 17. The drugs were intraperitoneally administered once every two days when the tumor volume reached to approximately 200 mm^3^. Tumor size and mice body weight were monitored up to 4 weeks and tumor volume (TV) was calculated by the above formula.

### Flow cytometry

SMMC7721 and PLC8024 cells were treated with sorafenib or 3-HAA at appropriate doses for 48 h and harvested by trypsinization and washed with phosphate buffered saline (PBS). The cells were then stained with anti-human Annexin V-FITC and PI-PE (#: AP107-100, Multisciences Inc, Shanghai, China) for 30 min. At least 1 x 10^6^ cells were analyzed by a FACS Aria II (BD Falcon, Franklin Lakes, NJ, USA). Cells were gated based on their forward and side scatter properties. Furthermore, mouse xenograft tissues were minced and digested with collagenase and DNAses (Life Technologies, Grand Island, NY, USA) at 37 °C for 30 min, and then filtered with 40-micron cell strainers (BD Falcon) Tumor cells were stained with annexin-V/propidium and analyzed by flow cytometry.

### Apoptotic assays

TUNEL assays were performed using commercially available kit (Roche, Basel, Switzerland) as instructed by the manufacturer. Adherent cells and OCT-embedded tumor tissue sections were fixed with 4% paraformaldehyde. After permeabilization with 0.1% Triton X-100 in PBS for 10 min, slides were incubated with methanol containing 3% H_2_O_2_, followed by dUTP nick end labeling at 37 °C for 1 h. The prolong gold antifade mounting solution containing 40, 6-diamidino-2-phenylindole (DAPI) (Invitrogen) was added to tissue sections prior to coverslip mounting.

### ChIP-Sequencing analysis

Chromatin was isolated from HCC cells treated with or without 3-HAA and fragmented to a size range from 150 to 400 bp. The solubilized chromatin fragments were immunoprecipitated with antibodies against YY1. The recovered DNA fragments were processed for DNA sequencing by the Illumina Genome Analyzer. The generated short reads were mapped onto the genome, and the peak calling program was used to identify peaks with the mapped reads.

### Western blotting assays

Appropriate cells were lyzed in RIPA lysis buffer containing a cocktail of protease inhibitors (Roche) and PMSF. Total protein concentration was determined using the bicinchoninic acid (BCA) assay kit (Ding Guo Biotechnology, Shanghai, China). Antibodies against the following proteins were used for immunoblotting: phospho-Akt (Thr308) (#2965S , dilutions 1:1000), phospho-Akt (Ser473) (#4060S, dilutions 1:1000), Akt (#4691L, dilutions 1:1000), phospho-PDK1 (Ser241) (#3438s, dilutions 1:1000), and PDK1 (#3062, dilutions 1:1000), phospho-GSK-3β (Ser9) (#9323P, dilutions 1:1000), GSK-3β (#9315L, dilutions 1:1000), β-catenin (#8480, dilutions 1:1000), phospho-p44/42 MAPK (Erk1/2) (Thr202/Tyr204) (#4370S, dilutions 1:1000), p44/42 MAPK (Erk1/2) (#4695S, dilutions 1:1000), cleaved caspase 3 (#9315L, dilutions 1:1000), all from Cell Signaling Technology (MA, USA). PARP1 (#13371-1-AP, dilutions 1:1000), PPP1R15A (#10449-1-AP, dilutions 1:500), (ProteinTech, Rosemont, IL, USA); PPP1α (#271762, dilation 1:1000) and β-actin (#47778, dilutions 1:2000), (Santa Cruz Biotechnology, Santa Cruz, CA, USA); YY1 (#61779, dilutions 1:500, Active Motif, CA, USA). The immunoblots were scanned using an Odyssey infrared imaging system (LI-COR). Immunolabeling was detected using the ECL reagent (Sigma). Protein expression was normalized against β-actin.

### *In vitro* Phosphatase assay

The activity of immunoprecipitated PPP1R15A/PPP1α was determined by using phosphorylated AKT proteins as the substrate. Specifically, SMMC7721 cells were treated with 3-HAA for 24 h. To immunoprecipitate the PPP1R15A/PPP1α proteins, 1 mg of cell lysate was mixed with 4 µg of mouse anti-PPP1R15A antibody, incubated at RT for 6 hrs, followed by the addition of 15 µl of protein A/G-agarose beads and rotated at RT for 1 hr. Pelleted beads were washed twice with PPP1 buffer [50 mM Tris (pH 7.0), 0.1 mM EDTA, and 1 mM MnCl2]. Forty µl of PPP1 buffer was added to each tube, containing 5 mM DTT, 100 ng of phosphorylated AKT protein (Upstate Biotechnology), with/without okadaic acid at the indicated concentration (Sigma). Tubes were incubated at 30 °C for 45 min, with occasional vortexing. The reaction was stopped by addition of 10 µl of 5 × SDS sample buffer. Phosphorylation of the AKT protein was detected by Western blotting with antiphospho-AKT (T308) antibody.

### Real-time quantitative PCR

Total cellular RNA was prepared using the TRIzol reagent (Invitrogen) as instructed by the manufacturer and was reverse transcribed using an RT reagent kit (TAKARA, Dalian, China). After cDNA synthesis, real-time quantitative polymerase chain reaction (PCR) was performed in triplicate in a 96-well plate with an ABI7500 real-time PCR system (Life Technologies, Grand Island, NY, USA) using SYBR Green mixture. CTNNB1 expression was normalized against β-actin. The primer sequences were as follows: *CTNNB1*, forward 5'-ATGATGGTCTGCCAAGTGGG-3' and reverse 5'-GGCCATCTCTGCTTCTTGGT-3' and β-actin, forward 5'-GCGGGAAATCGTGCGTGACATT-3' and reverse 5'-GATGGAGTTGAAGGTAGTTTCG-3'.

### Statistical analysis

Data were presented as means ± SD. All data were representative of at least three independent experiments. Differences between groups were assessed by Student's *t* test; all presented differences were *P* < 0.05 unless otherwise stated.

## Results

### AKT is activated in sorafenib-resistant HCC cells

**Figure 1:**
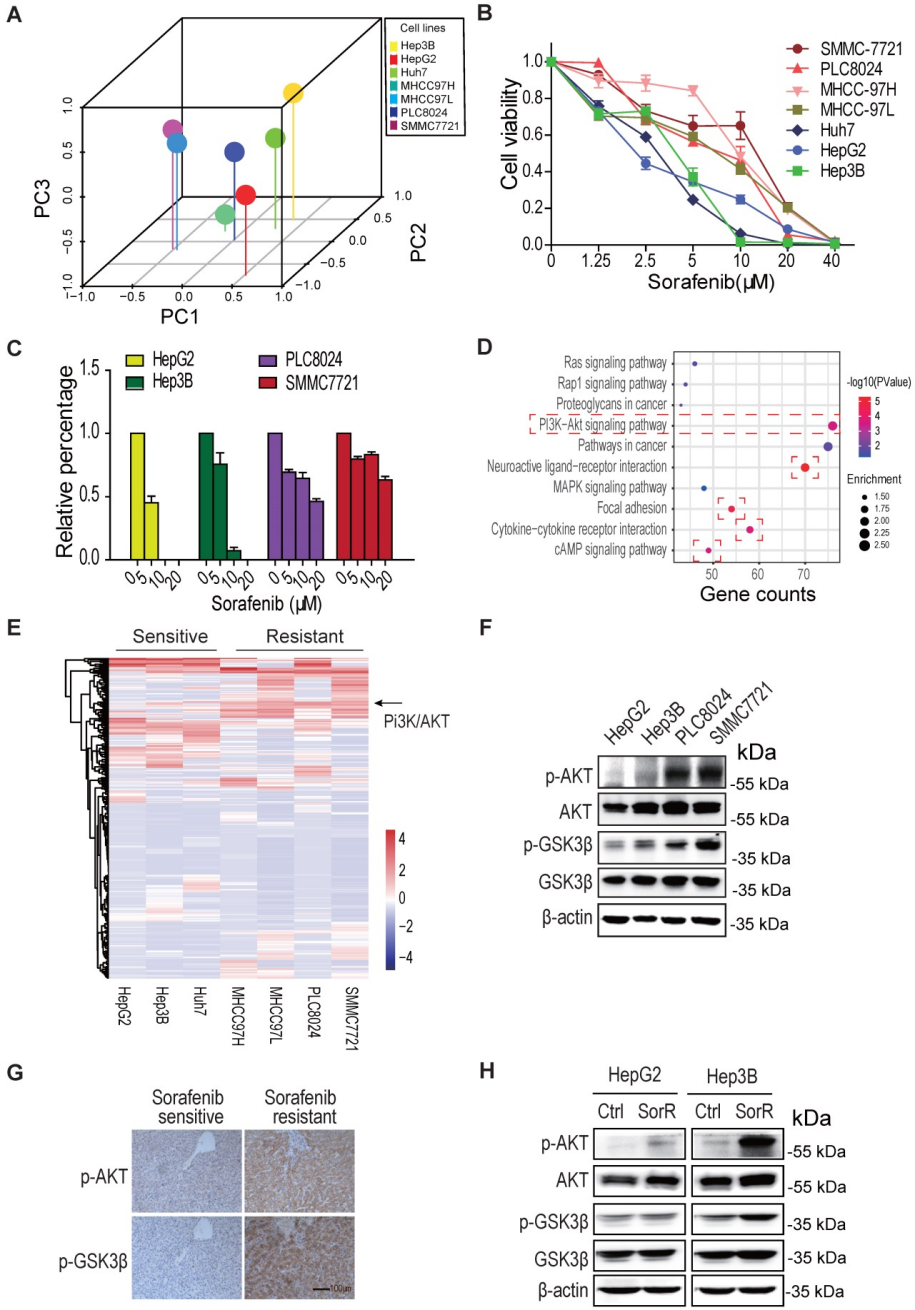
** AKT is activated in sorafenib-resistant hepatocellular carcinoma (HCC). A.** The principal component analysis (PCA) on various HCC cells. **B & C.** SMMC7721 and PLC8024 cells are more resistant to sorafenib than HepG2 and Hep3B cells. HCC cells were treated with sorafenib as detailed in Methods and viability was analyzed by the CCK8 method (B) and colony formation assays (C). Data shown are mean ±SD of at least 3 independent experiments. **D.** The gene ontology analysis on the upregulated/downregulated genes. The RNA-sequencing were performed, the genes whose expression changed more than ± 2 folds in sorafenib-resistant HCC cells were analyzed, compared to sorafenib-sensitive HCC cells. The PI3K/AKT, focal adhesion, cytokine-cytokine receptor, neuroactive ligand-receptor, and cAMP signaling were activated in *de novo* sorafenib-resistant PLC8024 and SMMC7721 cells. **E.** The heatmap display of upregulated/downregulated genes among sorafenib-sensitive and sorafenib-resistant HCC cells. The change folds of gene expression were more than ± 2. **F.** Akt was phosphorylated in *de novo* sorafenib-resistant PLC8024 and SMMC7721 cells. The T308 phosphorylation of Akt and S9 phosphorylation of GSK3β were analyzed. **G.** Akt was phosphorylated in sorafenib-resistant HCC tumors. Tumor sections for phospho-AKT and phospho-GSK3β staining in each set were from adjacent slices of same tumor. **H.** AKT was phosphorylated in acquired sorafenib-resistant HepG2 and Hep3B cells.

To investigate the mechanism by which HCC cells possess *de novo* or acquired resistance to sorafenib, the gene expression profiling from various type of HCC cells was analyzed by the principal component analysis (PCA). As shown in Fig. 1A, the distributions of HepG2, Hep3B, and Huh7 are different from the MHCC97H, MHCC97L, PLC8024, and SMMC7721 in the 3D graph, indicating the two groups of cells may have distinct properties. Both cell survival assay and colony formation assay displayed their difference in sorafenib sensitivity, SMMC7721 and PLC8024 cells were more resistant to sorafenib than HepG2 and Hep3B HCC cells to sorafenib (Fig. 1B, 1C & S1A).

The gene ontology analysis on the common upregulated/downregulated genes between the two groups of HCC cells displayed that PI3K/Akt, focal adhesion, Neuroactive ligand/receptor, cytokine/cytokine receptor, and cAMP signaling pathways were mostly regulated in sorafenib-resistant cells (Fig. 1D). The heatmap of gene expression in two groups of cells showed that the PI3K/Akt signal was dramatically activated in three sorafenib-resistant cells (Fig. 1E &S1B).

Moreover, both the immunoblot analysis and immunochemistry staining demonstrated that the T308 phosphorylation of Akt was significantly increased not only in *de novo* sorafenib-resistant HCC cells and tissues (Fig, 1F, 1G & S1C) but also in acquired sorafenib-resistant HCC cells (Fig. 1H & S1D). These observations supported the AKT activation leads to HCC sorafenib resistance.

### 3-HAA sensitizes HCC cells to sorafenib both* in vitro and in vivo*

**Figure 2:**
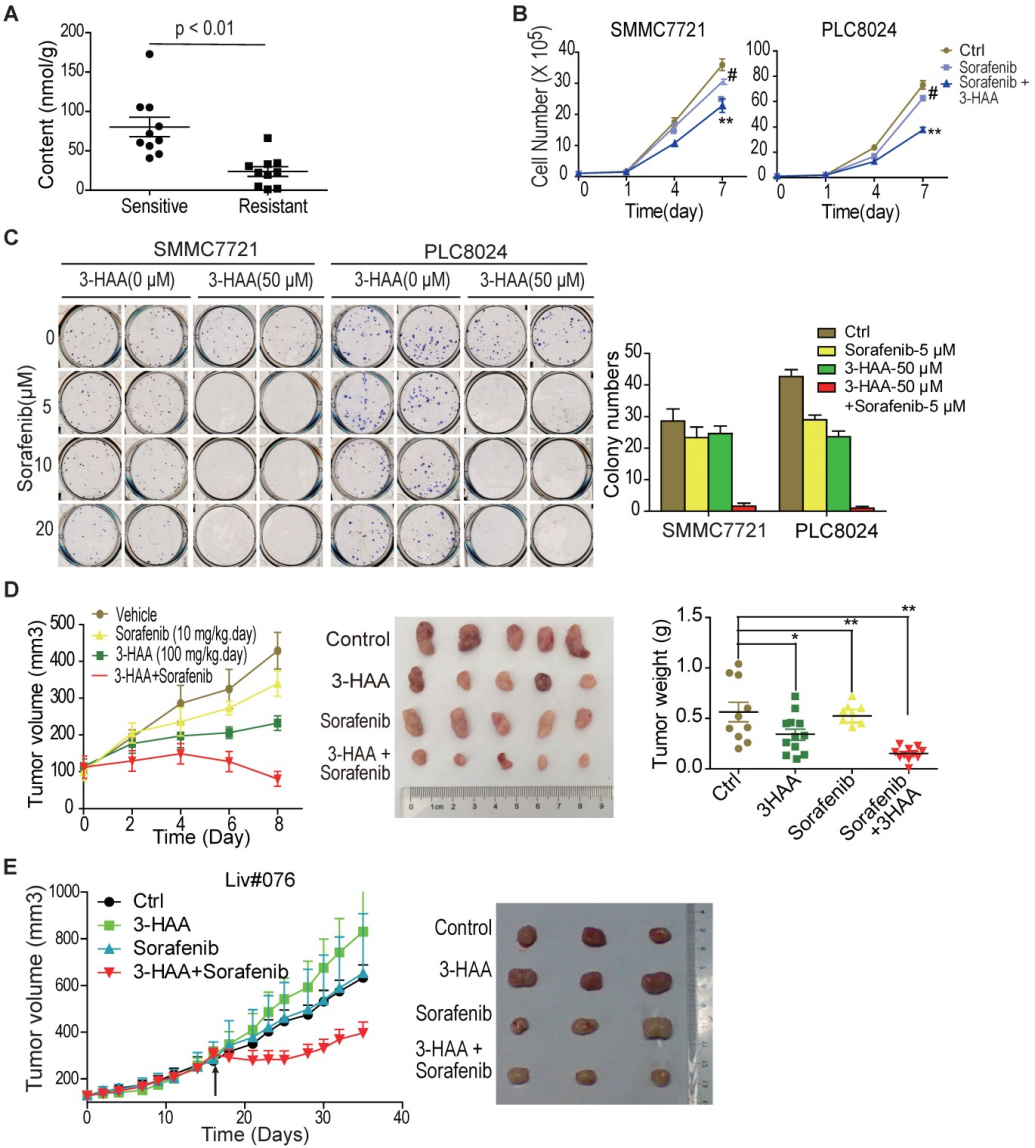
** 3-HAA sensitizes HCC to sorafenib both *in vitro* and *in vivo.* A.** Quantitative analysis of 3-HAA by LC-MS/MS in sorafenib sensitive or resistant HCCs. **B.** 3-HAA inhibits cell growth of sorafenib-resistant HCC cells. The cell numbers of HCC cells were counted by cytometry. Data shown are mean ± SD of at least 3 independent experiments. **C.** 3-HAA reduces colony formation in sorafenib-resistant SMMC7721 and PLC8024 cells. The cells were treated with sorafenib at the indicated dose with and without 50 μM of 3-HAA for 4 days. The graphic shows colony numbers as cells treated with 5 μM of sorafenib with or without 50 μM of 3-HAA. Data shown are mean ± SD of at least 3 independent experiments. **D.** The combination of 3-HAA and sorafenib synergistically suppresses the growth of SMMC7721 xenografts. 3-HAA and the sorafenib were administered by intraperitoneal injection for 7 days at the dose of 100 mg/kg.day and 10 mg/kg.day, respectively. Tumor volumes and tumor weight are presented as mean ± SD (*: P < 0.05; **: P < 0.01.). Five mice were recruited in each group. **E.** The combined treatment with 3-HAA dramatically suppresses tumor growth of patient-derived sorafenib-resistant HCC xenografts. As xenografts reached at approximately 250 mm^3^, 3-HAA and the sorafenib were administered by intraperitoneal injection for 14 days at the dose of 100 mg/kg.day and 30 mg/kg.day, respectively. Three mice were recruited in each group.

Our data showed 3-hydroxyanthranilic acid (3-HAA), a structural analog of kynurenine, was reduced in HCC cells and exogenous 3-HAA enhanced apoptosis in HCC (Shi, et al. Kynurenine derivative 3-HAA is an agonist ligand for transcript factor YY1). We were interested in whether 3-HAA sensitized HCC cells to sorafenib. The cellular concentration of 3-HAA was first analyzed in sorafenib sensitive or resistant HCCs. The GC-MS/MS data showed that 3-HAA concentration decreased in sorafenib resistant HCCs, compared to sorafenib sensitive HCCs (Fig. 2A).

The response of sorafenib-resistant HCC PLC8024 and SMMC7721 cells to 3-HAA was then determined by CCK8 assays and colony formation assays. As shown in Fig. S2A, 3-HAA inhibited HCC cell growth in both dose- and time- dependent manner. Colony formation assays demonstrated inhibitory effects of 3-HAA at the indicated doses (Fig. S2B). Moreover, both the cytometry assays and the colony formation assays showed that the combination of sorafenib and 3-HAA at the indicated doses reduced HCC cell numbers and colony numbers (Fig. 2B & 2C). The graphic showed that the addition of 50 μM 3-HAA to 5 μM sorafenib caused greater colony number reduction of SMMC7721 and PLC8024 cells than either agent alone (Fig. 2C).

Tumor xenograft studies additionally revealed that sorafenib (10 mg/Kg.day) plus 3-HAA (100 mg/Kg.day) caused growth delay of mouse HCC xenografts, with marked reduced tumor volume and tumor weight (Fig. 2D), without affecting body weights and renal/liver function of mice (Fig. S2C&S2D). The growth inhibitory effect of sorafenib (30 mg/Kg.day) plus 3-HAA (100 mg/kg.day) was further confirmed in a patient-derived HCC xenograft model (Liv#076) (Fig. 2E), although sorafenib or 3-HAA alone might not significantly affect tumor growth at the applied dose.

These aforementioned results demonstrated that 3-HAA was closely correlated with HCC progression and exogenous 3-HAA sensitized HCC cells to sorafenib both *in vitro* and *in vivo*, highlighting the promise of 3-HAA as a potential drug for HCC therapy.

### The combined treatment with 3-HAA induces apoptosis of sorafenib-resistant HCC cells by inhibiting AKT

**Figure 3:**
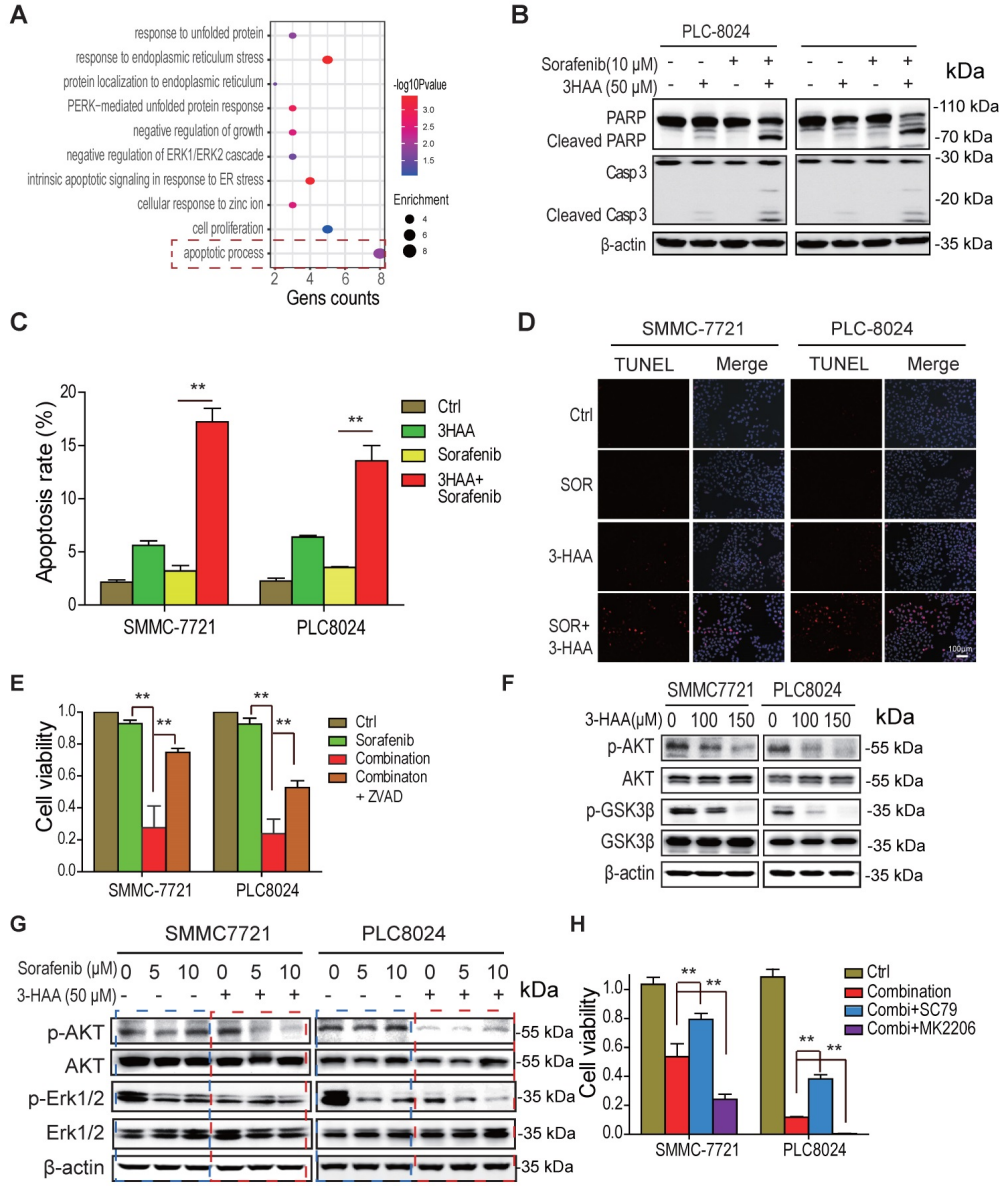
** The combined treatment with 3-HAA increases apoptosis of sorafenib -resistant HCC cells by inhibiting AKT. A.** the gene ontology analysis on the upregulated/downregulated genes. The RNA-sequencing were performed, the genes whose expression changed more than ± 2 folds in combination-treated HCC cells were analyzed, compared to only sorafenib-treated HCC cells. **B.** The levels of cleaved caspase 3 and cleaved PARP were analyzed in the sorafenib- and/or 3-HAA-treated SMMC7721 and PLC8024 cells. Cells were treated at the indicated dose for 24 h. **C. & D.** The apoptotic population were analyzed by flow cytometry (C) and TUNEL assay (D) in the sorafenib- and/or 3-HAA-treated SMMC7721 and PLC8024 cells. **E.** The combination of 3-HAA and sorafenib dramatically reduces cell viability of SMMC7721 and PLC8024 cells. The treating time was 14 days. Cells were treated with the 5 μM of sorafenib alone, or the combination with the 50 μM of 3-HAA. **F.** The effect of 3-HAA on Akt activity in sorafenib-resistant HCC cells. **G.** The Akt activity were analyzed in HCC cells treated with sorafenib alone, or the combination with the 50 μM of 3-HAA for 24 h. **H.** The AKT activator (SC79) attenuates while AKT inhibitor (MK2206) enhances the combination-induced cell death. The histogram shows the relative viability of HCC cells. The concentration of SC79 and MK2206 was 15 μM and 10 μM, respectively. The cells were treated with the combination of 5 μM of sorafenib and 50 μM of 3HAA for 4 days.

To explore the signaling suppressing cell growth of sorafenib-resistant HCC cells in response to the combination of sorafenib and 3-HAA, the gene ontology analysis was performed on the differential expressed genes of SMMC7721 or PLC8024 cells treated by sorafenib and 3-HAA. The sorafenib alone-treated cells were considered as a control. As shown in Fig. 3A, the most activated signal was the apoptotic process pathway, suggesting the combination with 3-HAA induces apoptosis in sorafenib-resistant HCC cells.

Moreover, the immunoblots showed that the level of cleaved PARP and cleaved caspase 3 was dramatically increased in PLC8024 and SMMC7721 treated with sorafenib and 3-HAA (Fig. 3B). The flow cytometry analysis of SMMC7721 and PLC8024 cells displayed that the combination of sorafenib and 3-HAA significantly increased the population of apoptotic cells up to 18% (Fig. 3C & S3A). The TUNEL assay also confirmed that sorafenib with 3-HAA induced abundant apoptosis in sorafenib-resistant HCC cells (Fig. 3D). Also, the apoptosis inhibitor ZVAD restored the cell growth suppressed by the sorafenib and 3-HAA (Fig. 3E), suggesting that sorafenib with 3-HAA sensitize sorafenib-resistant HCC cells by enhancing apoptosis.

Since Akt signal is a master regulator for cell survival and apoptosis and highly activated in sorafenib-resistant HCC cells, we proposed that 3-HAA inactivates Akt to induce apoptosis. As shown in Fig. 3F & S3B, 3-HAA but sorafenib inhibited the Thr 308 phosphorylation of Akt in a dose dependent manner. The combination of sorafenib and 3-HAA dramatically reduced the Thr308 phosphorylation of Akt in both SMMC7721 and PLC8024 cells (Fig. 3G). Moreover, the Akt activator SC79 restored the cell viability suppressed by the combination of sorafenib and 3-HAA while the Akt inhibitor MK2206 further decreased the cell viability (Fig. 3H & S3C), suggesting that 3-HAA induces apoptosis of sorafenib-resistant HCC cells via inhibiting Akt activity.

### 3-HAA inhibits AKT activity by upregulating PPP1R15A

**Figure 4:**
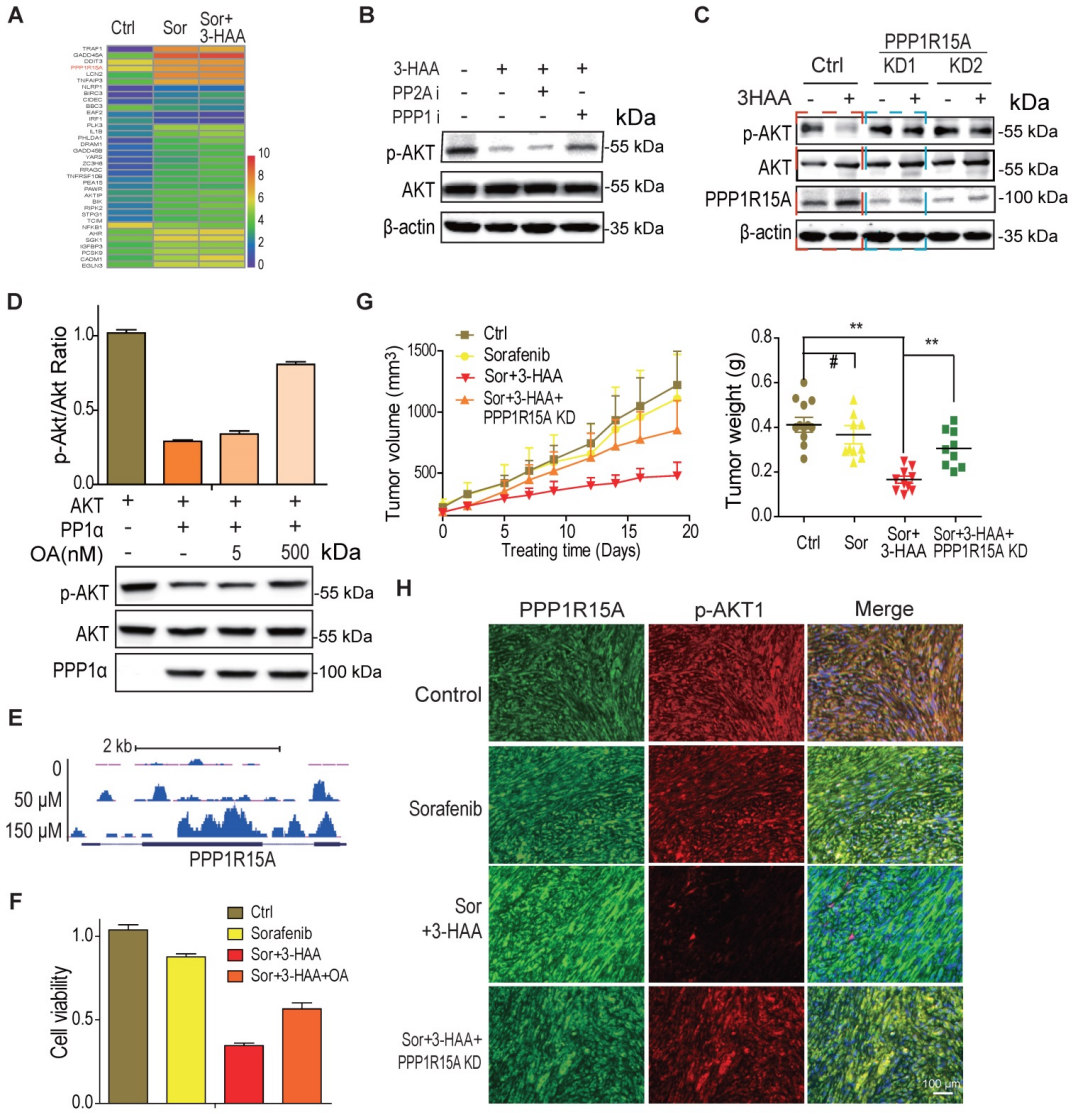
** 3-HAA inhibits AKT activity by upregulation of PPP1R15A. A.** The genes involved in apoptosis pathway and upregulated by the combination of sorafenib and 3-HAA. The concentration of sorafenib and 3-HAA were 5 μM and 50 μM, respectively. **B.** The phosphatase PPP1α inhibitor okadaic acid but PPP2A inhibitor Calyculin A restored the AKT phosphorylation in 3-HAA-treated SMMC7721 cells. The dose of both calyculin A and okadaic acid were 2 nM. Cells were treated for 4 h. **C.** The PPP1R15A knockdown restores AKT phosphorylation suppressed by 3-HAA. The treating time and 3-HAA dose were the same as above. **D.** The effects of PPP1R15A/PPP1α on Akt phosphatase. The phosphatase assay was described in methods. **E.** The ChIP-sequencing analysis of YY1 on PPP1R15A gene. **F.** PPP1α inhibitor Okadaic acid (OA) partially restored the cell survival. The dose of Okadaic acid and the 3-HAA was 2 nM and 100 μM, respectively. Cells were treated for 24 h. **G.** The combination of sorafenib and 3-HAA dramatically decreased the tumor growth and tumor weights while PPP1R15A knockdown restored the xenograft growth and xenograft weights. Five mice were recruited in each group. **H.** The combination with 3-HAA treatment upregulated PPP1R15A expression and inhibited Akt activation in SMMC7721 xenografts. As xenografts reached at approximately 100 mm^3^, the sorafenib and/or 3-HAA were administered by intraperitoneal injection for 14 days at the dose of 100 mg/Kg.day and 30 mg/Kg.day, respectively.

To explore the mechanisms whereby 3-HAA inhibits Akt activity in sorafenib-resistant HCC cells, the genes involved in the apoptosis process and upregulated by the combination treatment were displayed in the heatmap. As shown in Fig. 4A, the PPP1R15A, a regulatory subunit of phosphatase PPP1α and also known as GADD34, was dramatically upregulated by the combination of sorafenib and 3-HAA in sorafenib-resistant HCC cells. And, the PPP1 inhibitor (okadaic acid, OA, 2nM) but not the PPP2A specific inhibitor (calyculin A, 2nM) recovered the Thr 308 phosphorylation of Akt (Fig. 4B), indicating phosphatase(s) may involve in Akt inactivation/dephosphorylation. Moreover, the PPP1R15A knockdown but not PPP2Ac knockdown restored the 3-HAA-reduced Akt phosphorylation (Fig. 4C & S4). The phosphatase assay further confirmed that PPP1α dephosphorylated Akt, and its inhibitor OA diminished its effects in a dose-dependent manner (Fig. 4D).

Our data demonstrated that 3-HAA binds transcription factor YY1 to regulate a broad range of genes (Shi, et al. Kynurenine derivative 3-HAA is an agonist ligand for transcript factor YY1). Here, our YY1 ChIP-sequencing analysis showed that YY1 indeed enriched to the promoter and gene body of *PPP1R15A* in a 3-HAA dose-dependent manner (Fig. 4E). This observation supported our finding of 3-HAA upregulation of PPP1R15A.

The function studies also showed that the PPP1 inhibitor OA recovered the cell viability suppressed by the combination of sorafenib and 3-HAA (Fig. 4F) and PPP1R15A knockdown restored the HCC xenograft growth inhibited by the combination of sorafenib and 3-HAA, evidenced by the tumor growth curve and the final tumor weight (Fig. 4G). The immunofluorescence staining data showed that the Thr308 phosphorylation of Akt also restored in PPP1R15A knockdown xenografts (Fig. 4H), suggesting 3-HAA inhibits AKT activity/phosphorylation by upregulating PPP1R15A.

### 3-HAA-upregulated DUSP6 also diminishes Akt activity by dephosphorylating PDK1

**Figure 5:**
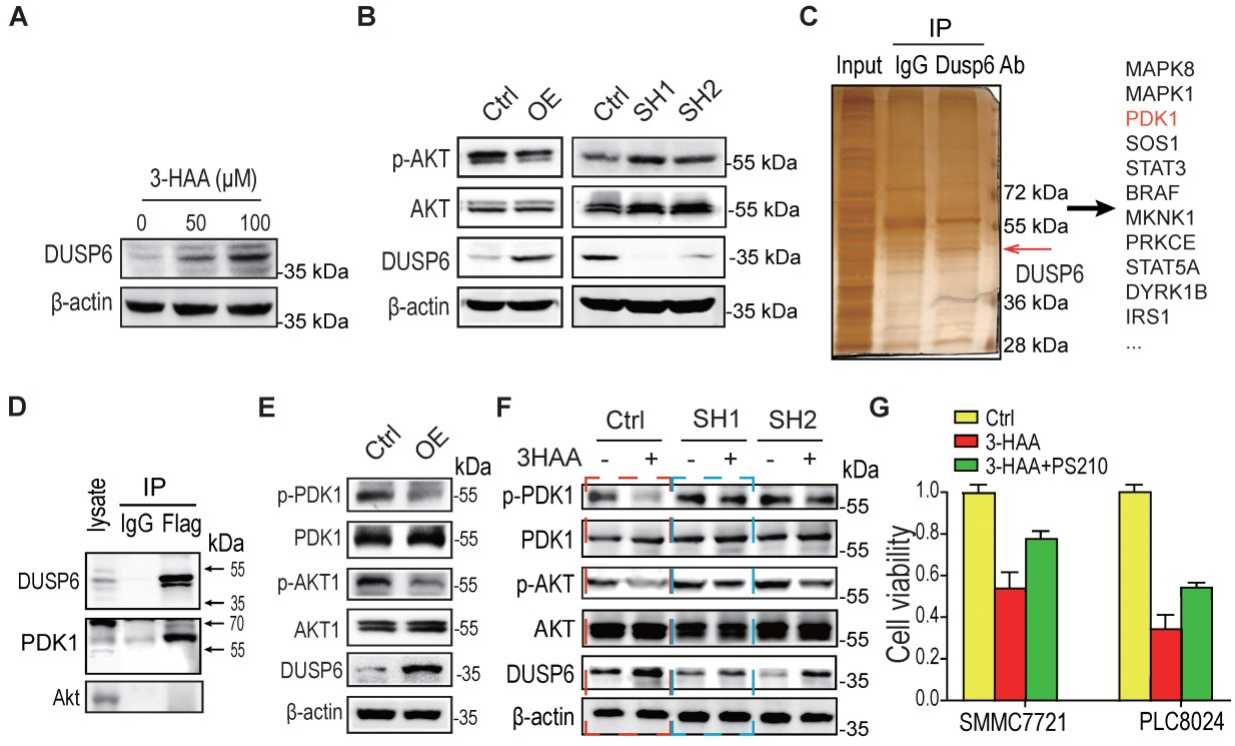
** 3-HAA-upregulated DUSP6 also diminishes Akt activity by dephosphorylating PDK1. A.** DUSP6 expression detection in SMMC7721 cells treated with 3-HAA for 24 h at the indicated dose. **B.** The effect of DUSP6 on the Akt activity. T308 phosphorylation of Akt was detected in SMMC7721 cells overexpressing DUSP6 or depleted of DUSP6. **C.** Detection of DUSP6 binding proteins by mass spectrometry following co-immunoprecipitation. **D.** Detection of DUSP6 in PDK1-associated proteins. PDK1 was tagged with flag. **E.** The effect of DUSP6 overexpression on PDK1 and Akt activity. PDK1 phosphorylation (S241) and AKT phosphorylation (T308) were detected separately. **F.** DUSP6 knockdown fully restored 3-HAA-reduced PDK1 phosphorylation (S241) and partially recovered 3-HAA-deminished AKT phosphorylation (T308). The SMMC7721 cells were treated with 100 μM of 3-HAA for 24 h. **G.** PDK1 activator (PS210) rescued 3-HAA-induced cell death. The dose of PS210 was 10 μM. All cells were treated with 5 μM sorafenib for 4 days. The histogram shows the relative viability of HCC cells treated with 100 μM of 3HAA with or without 10 μM PS210.

Through the above expression profiling analysis, the other phosphatase DUSP6 besides the PPP1R15A was also significantly increased in the combination-treated sorafenib-resistant HCC cells (SMMC7721 cells and PLC8024 cells), compared to the sorafenib alone-treated these cells (Fig. 5A). Moreover, overexpression of DUSP6 reduced Akt phosphorylation while the depletion of DUSP6 increased Akt phosphorylation (Fig. 5B), suggesting that the phosphatase DUSP6 regulates Akt activity.

To explore the mechanism by which DUSP6 regulating Akt activity, the co-immunoprecipitation against DUSP6 was performed. As shown in Fig. 5C, several protein kinases including PDK1 were co-immunoprecipitated with DUSP6. The immunoblotting analysis further confirmed that DUSP6 bound to PDK1 (Fig. 5D). Moreover, PDK1 activity was decreased in SMMC7721 cells overexpressing DUSP6, reflected by the PDK1 phosphorylation at Ser 241 (Fig. 5E). Although the knockdown of DUSP6 did not increase the basal phosphorylation level of PDK1/Akt too much, it's knockdown fully restored the 3-HAA-reduced PDK1 phosphorylation, and partially recovered the 3-HAA-deminished Akt phosphorylation, which was consistent with our above finding (Fig. 5F) and the previous report 18. The cell growth rate indicated by cell numbers further showed that the PDK1 activator PS210 partially recovered the 3-HAA-suppressed HCC cell growth (Fig. 5G), suggesting DUSP6 also regulates 3-HAA reducing Akt activity through dephosphorylating PDK1.

### AKT inhibition is critical for 3-HAA sensitization of HCC to sorafenib

**Figure 6:**
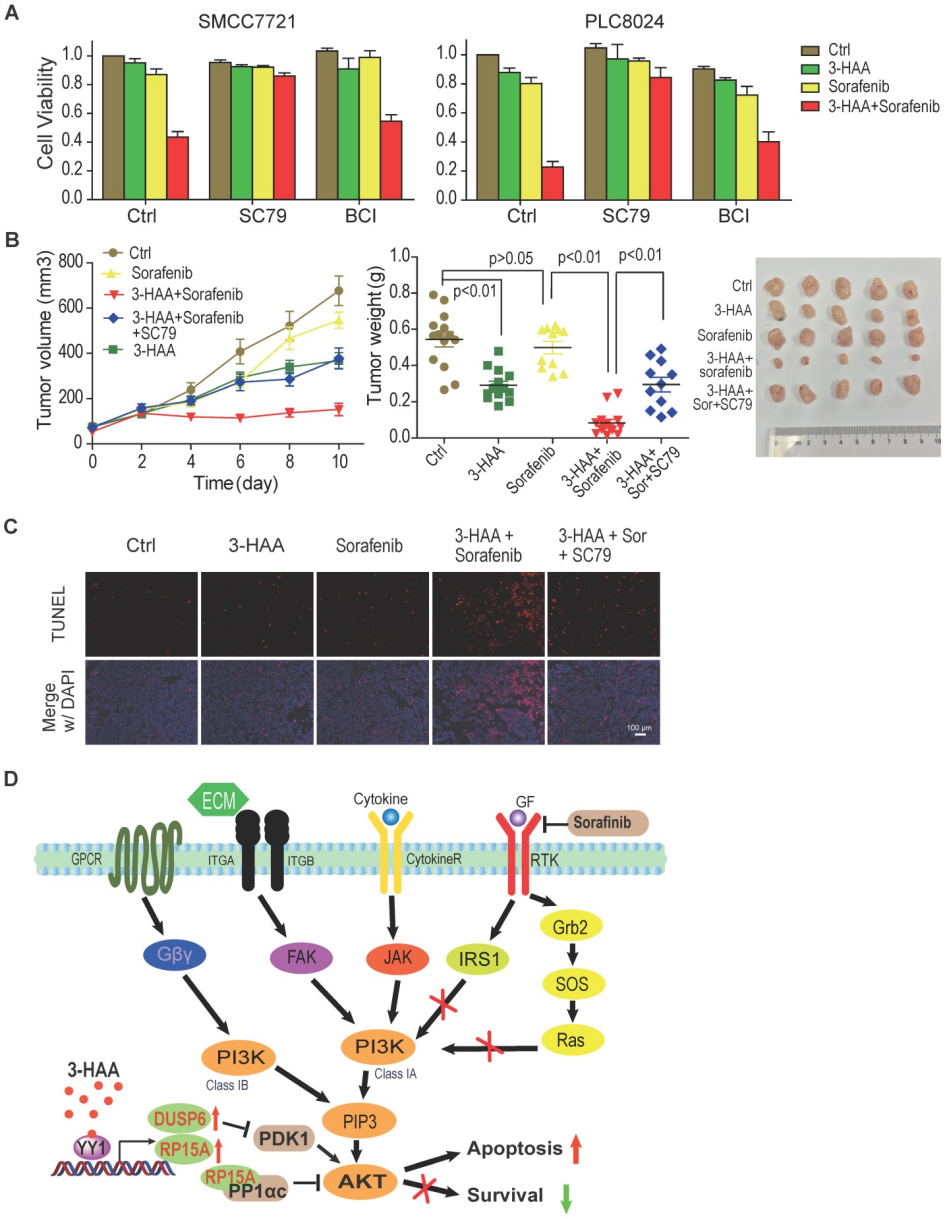
** AKT inhibition is critical for 3-HAA sensitization of HCC to sorafenib. A.** The Akt activator SC79 restored HCC cell growth inhibited by the combination of 3-HAA and sorafenib while the ERK activator BCI partially restored HCC cell growth. Cell viability was examined by the CCK8 method. The final concentration of sorafenib and 3-HAA was 5 μM and 50 μM, respectively. The final concentration of SC79 and BCI was 15 μM and 10 μM, respectively. **B.** The SC79 recovered tumor growth of SMMC7721 xenografts suppressed by the combination treatment. The tumor volumes are presented as mean ± SD. The dose of 3-HAA and sorafenib was 100 mg/Kg.day and 10 mg/Kg.day, respectively. The dose of SC79 was 40 mg/Kg.day. Five mice were recruited in each group. **C.** The apoptosis detection in xenografts by TUNEL assay. **D.** The working model for 3-HAA sensitizing sorafenib-resistant HCC cells. 3-HAA-upregulated PPP1R15A/PPP1α dephosphorylate/inactivates Akt which was compensatorily reactivated in sorafenib-resistant HCC cells, and consequently sensitized HCC cells to sorafenib.

To further determine whether Akt inhibition was indispensable for the synergistic effect of 3-HAA and sorafenib on HCC, we evaluated the effect of Akt and ERK activator on cell viability in SMMC7721 and PLC8024 cells treated with sorafenib plus 3-HAA. We found that sorafenib plus 3-HAA significantly inhibited cell viability of SMMC7721/PLC8024, and the Akt activator SC79 almost fully recovered the cell viability decreased by sorafenib plus 3-HAA while the ERK activator BCI only partially recovered the cell viability although BCI dramatically increased ERK phosphorylation (Fig. 6A & S5A).

Furthermore, Akt activator SC79 abolished the synergistic effect of 3-HAA and sorafenib on HCC mouse xenografts growth (Figure 6B), without affecting mouse body weight (Fig. S5B). The apoptosis analysis of xenografts by TUNEL assay showed sorafenib and 3-HAA markedly increased the apoptotic cell population, evidenced by the TUNEL foci, and AKT activator SC79 nearly diminished these foci (Fig. 6C), suggesting the combination of 3-HAA and sorafenib increase apoptosis by inhibiting Akt activity.

## Discussion

Cancer cells tend to develop resistance upon repeated exposure to anticancer drugs and certain cancers are also constitutively resistant. The discovery of sorafenib provided hope for combating HCC, but this promising treatment only demonstrated limited survival benefits, reflected by a low response rate to sorafenib (*de novo* resistance) and quick recurrence after initial response (acquired resistance)^5^. Unfortunately, there are no available alternative effective drugs against HCC currently 19. Therefore, it is necessary to develop novel therapeutic agents that can overcome acquired or *de novo* resistance to sorafenib that could lead to the discovery of promising strategies to increase therapeutic efficacy against HCC.

The current study demonstrates that 3-HAA, a structural analog of kynurenine, sensitizes HCC cells to sorafenib both* in vitro* and *in vivo*. Thus, the endogenous 3-HAA level is downregulated in sorafenib - resistant HCC cells, possibly due to the downregulation of kynurenine 3-monooxygenase (KMO) and kynureninase (KYNU), enzymes converting kynurenine into 3-HAA, and/or the upregulation of hydroxyanthranilate 3,4-dioxygenase (HAAO), enzyme degrading 3-HAA. Consequently, the newly generated kynurenine is secreted into the extracellular matrix to promote tumor immune evasion.

This tumor-growth inhibitory effect of 3-HAA is at least partially attributed to its induction of apoptosis of HCC cells via upregulating PPP1R15A (Figure 5D). PPP1R15A is also known as GADD34, recruiting PPP1 to dephosphorylate numerous targets 20, 21. Our findings provide the first proof of concept that adding an inhibitor of the kynurenine pathway to sorafenib therapy could lead to synergistic suppression of HCC growth both *in vitro* and *in vivo.* Indeed, our data have demonstrated that 3-HAA has a synergistic effect with sorafenib against HCC through the suppression of AKT activation.

Recent studies show that kynurenine promotes tumor progression and immune evasion through binding to the acyl hydrocarbon receptor (AHR) 15, 22, 23. However, our recent study demonstrates that 3-HAA directly binds to the transcription factor YY1 but AHR and exogenous 3-HAA induces apoptosis by inhibiting Akt and ERK activity, which is distinct from the effects of kynurenine, due to their different target. Previous studies demonstrated that PPP2A is an upstream serine/threonine protein phosphatase of AKT and dephosphorylates/inactivates AKT at Ser473 24, 25. Our data show that 3-HAA-upregulated PPP1R15A/PPP1 dephosphorylates/inactivates Akt at Thr308, which could result from the compensatory pathway of Akt activation in sorafenib-resistant HCC cells.

Taken together, our results demonstrate that 3-HAA promoted the sensitivity of HCC to sorafenib by suppressing the activity of AKT. 3-HAA-upregulation of PPP1R15A/DUSP6 inhibits AKT signaling and consequently increases the apoptosis of HCC cells (Fig. 6D). Besides, 3-HAA has a synergistic effect with sorafenib against HCC, suggesting that 3-HAA is a promising molecule for HCC therapy.

## Supplementary Material

Supplementary figures.
