# Supplementary material for: The kynurenine derivative 3-HAA sensitizes hepatocellular carcinoma to sorafenib by upregulating phosphatases
Source: Theranostics. 2021 Apr 3;11(12):6006–18. doi: 10.7150/thno.59841 (PMC8058709; doi:10.7150/thno.59841)
Supplement: Supplementary file 1 — Supplementary figures. [file thnov11p6006s1.pdf]

Figure S1

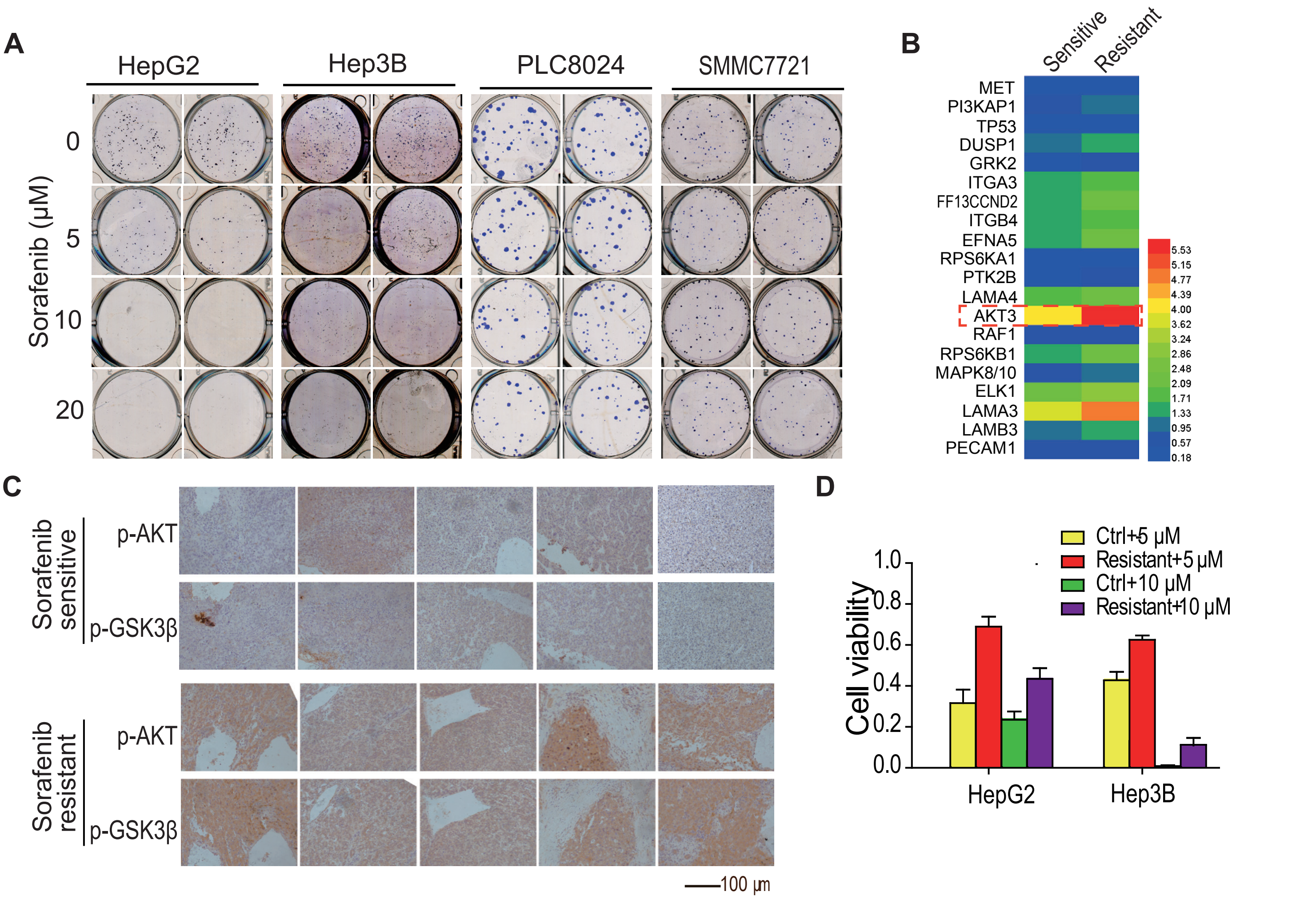

Figure S1 AKT is activated in sorafenib-resistant HCC cells

A. Colony formaton assay was analyzed in four cell lines treated with sorafenib at the indicated dose. The cells were grew for 14 days.

B. The proteins involved in the PI3K/AKT pathway and upregulated more than 1.5 folds in sorafenib-resistant HCC cells.

C. phospho-AKT and phospho-GSK3β detection by immunohistochemistry staining in clinical HCC samples.

D. Cell viability assay on acquired sorafenib-resistant HCC cells.

Figure S2

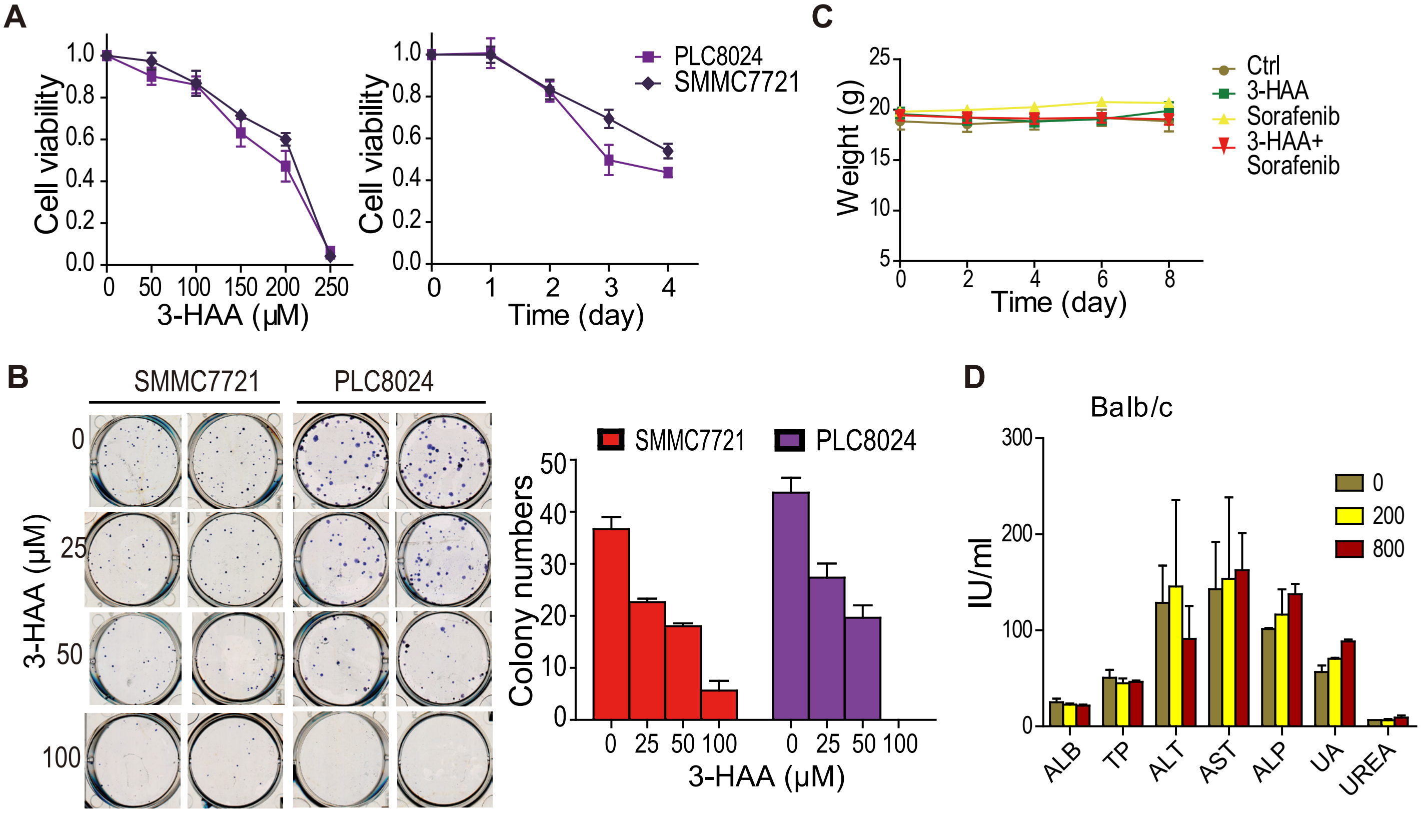

Figure S2 3-HAA sensitizes HCC to sorafenib both in vitro and in vivo.

A. 3-HAA inhibited sorafenib-resistant HCC growth.

B. The effects of 3-HAA on colomy formation of SMMC7721 and PLC8024 cells.

C. 3-HAA and sorafenib at the used dose had no significant effect on mice weight. The mice weight are presented as mean ± SD.

D. 3-HAA had no significant effects on renal and live functions of Balb/c mice.

Figure S3

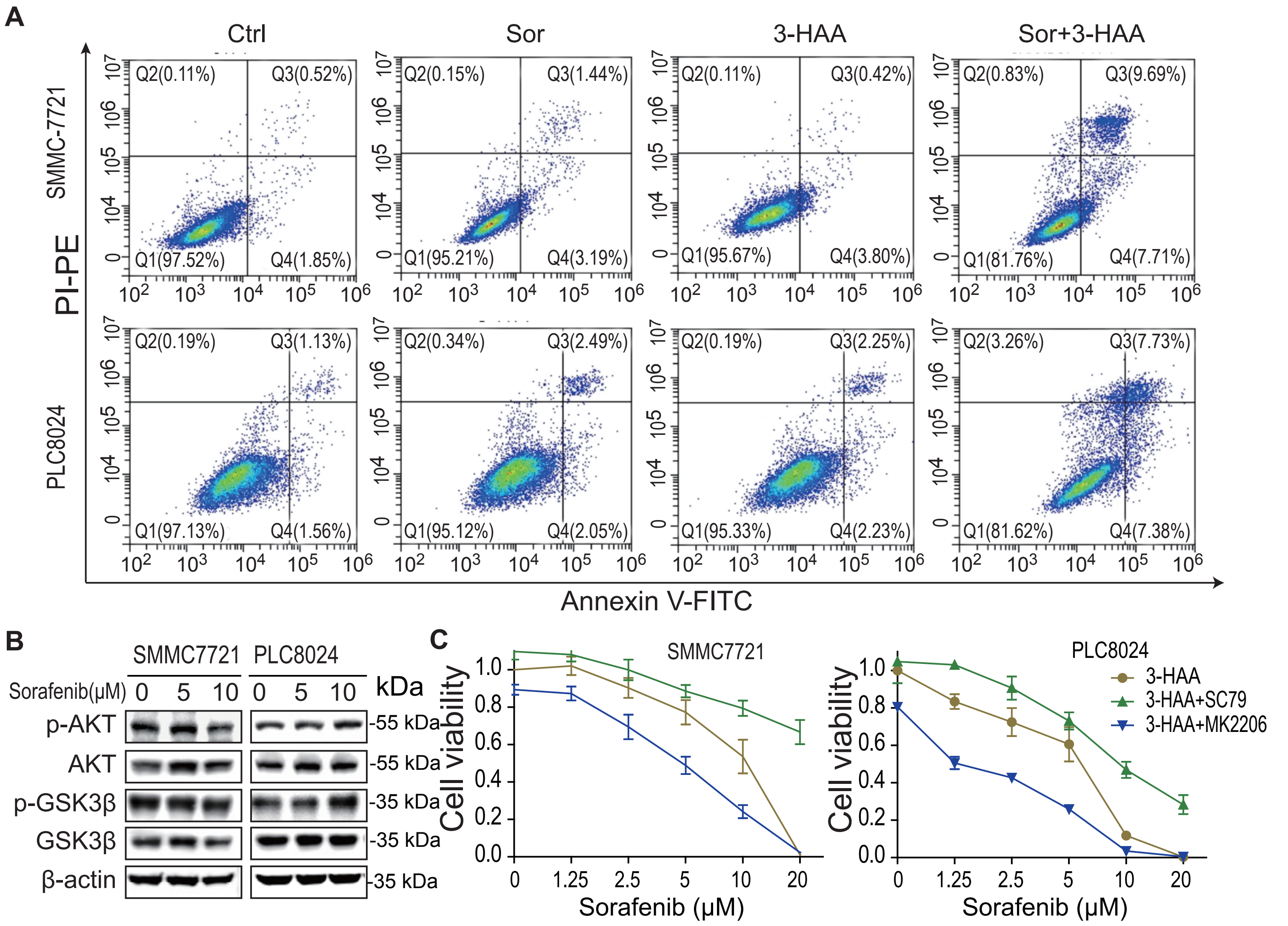

Figure S3 The combiend treatment with 3-HAA induces apoptosis of sorafenib-resistant HCC cells by inhibiting AKT

A. Effect of the combined treatment with 3-HAA on apoptosis detected by flow cytometry. Cells was treated for 24 hrs. The concentration of 3-HAA and sorafenib were 50 μM and 5 μM, respectively.

B. Effect of sorafenib on AKT activity in sorafenib-resistant HCC cells. Cells were exposed for 24 hrs.

C. Effect of the AKT inhibitor or activator on the efficacy of the combined treatment.

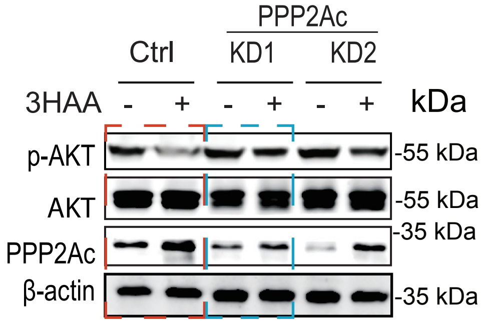

Figure S4 3-HAA inhibits AKT activity by upregulation of PPP1R15A

The effects of the PPP2Ac (PPP2A catalytic subunit) knock down on AKT activity.

Figure S5

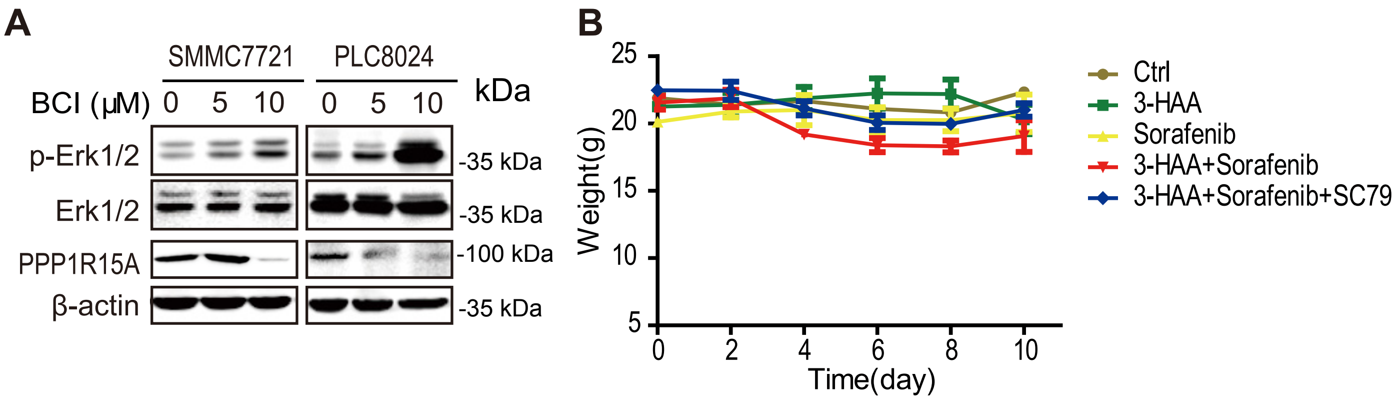

Figure S5 AKT inhibition is critical for 3-HAA sensitization of HCC to sorafenib

A. The BCI promoted ERK phosphorylation. The treating time was 24 h.

B. The 100 mg/Kg.day of 3-HAA, the 30 mg/Kg.day of sorafenib and the 40mg/Kg.day of SC79 did not have obvious influence on mice weight. The weight are presented as mean ± SD.
